# Annual Meeting of American Dental Association

**Published:** 1881-08

**Authors:** 


### TWENTY-FIRST ANNUAL MEETING OF THE AMERICAN DENTAL ASSOCIATION, HELD IN IRVING HALL, NEW YORK, July 12, 13, 14, 15, 1881.

OFFICERS, 1880 and 1881.

*President*, C. N. PIERCE; *1st Vice-President*, W. C. BARRETT; *2d Vice-President*, GEO. J. FREIDRICKS; *Recording Secretary*, GEO. N. CUSHING; *Corresponding Secretary*, A. M. DUDLEY; *Treasurer*, WM. H. GODDARD; *Executive Committee:* I. N. CROUSE, *Chairman;* C. D. STOCKTON, *Secretary*.

This body organized with eighty-nine members present. The President, Professor C. N. Pierce, of Philadelphia, called the association to order, and the Secretary, Dr. G. H. Cushing, of Chicago, read the minutes of the last annual meeting. The President stated that the Association had met a month earlier than usual so that those members who might wish to do so could have an opportunity of accepting the invitation which had been sent asking their attendance at an International Medical Congress, which is to take place in London next month (August). "It was to be regretted," the Professor went on to say, "that the medical profession had never appreciated the requirements of the dentist. Medical colleges every year were conferring degrees in dentistry, while the opthalmologist, the obstetrician, and the surgeon were all compelled to take a full course of medicine and receive the degree of M. D., before they could be recognized in any of the specialties. He thought that the practice of dentistry required just as careful an education as the branches of medicine did, and therefore inferred that the medical profession should give the subject more attention."

The President made some very practical remarks in the direction of the food we eat and its effect on the teeth and gums ; he believed that every practical and experienced dentist now before him would indorse him when he said that the teeth of those who lived upon dry, solid food were the most healthy, and those who subsisted largely on fluid and semi-fluid nourishment the reverse. Visits to the various institutions offered excellent opportunity for the examination of the teeth and for noting the influence upon them of the warm drinks which soften and wash the food into the stomach without mastication; his points were all well made; regarding the young miss and also the mother, he believed it quite possible for this body in a year or two to say to the public that there are certain physical, mental, moral and dietetic habits which are contributing both directly and indirectly in a large degree to the prevalence of teeth disease which is so destructive of health and beauty. Dr. W. H. Atkinson, Chairman of the Third Section, presented the report on nomenclature and terminology. The document was read by Mr. Stephen Pearl Andrews, of this city.

On the discussion of the filling of root cavity Drs. Atkinson, McKellops, of St. Louis, Wetherbee, of Boston, and Barrett, of Buffalo, led off. The sessions were fixed at 9 A. M. to 12 P. M., 2 P. M. to 5 P. M., 8 P. M. to adjournment.

"Operative Dentistry." This paper presented and read by Dr. George J. Freidricks, of New Orleans, La., was received with much attention. Drs. Mills, Brooklyn, Satch, of Philadelphia, Siles, of Boston, Merriman, of Salem, Mass., Webb, of Philadelphia, Schumway, of Plymouth, Mass, Crouse, of Chicago, Wetherbee, of Boston, gave their views and practical experiences in the same line. Dr. Siles' paper on the reflex action and shock in the dental specialty showed much research. "The Present Status of Histology," by Dr. Bodecker of this city, was a masterly production, and we regret our space forbids just here an extended notice. Next came Dr. Frank Abbott, of New York, with a carefully prepared paper on "Caries of the Human Teeth," the doctor supporting his position with large drawings prepared for the purpose. "Dr. Davenport, of Mass., reviewed the "Etiology of Chemical Abrasions of the Cutting Edges of the Front Teeth,"

Dr. Abbott's paper on "Caries of the Human Teeth" was discussed by Drs. Siles, Buckingham, Abbott, Friedricks, Mills Barrett, Moore, Chandler, Pierce, Waters, Crouse, Atkinson and Brophy. On motion of Dr. Abbott a committee of three was appointed to draft resolutions in reference to the shooting of President Garfield and expressive of the association's sympathy for himself and his family. The Chairman named as said committee Dr. Abbott, Dr. Litch, Dr. Friedricks.

Dr. F. M. Odell, who by request acted for Dr. Abbott as Chairman of this Committee, offered the following resolutions :

*Resolved*, That we sympathize profoundly with President James A. Garfield and his family in their present affliction.

*Resolved*, That we are utterly lacking in language sufficiently forcible to express our abhorrence of the attempt at his assassination and of its author.

*Resolved*, That it is our fervent prayer that in President Garfield we may continue to have the living example of a noble man who has so bravely borne up under difficulties.

The resolutions were unanimously approved and adopted.

Dr. Mills said from information received from a general in the army there is a movement on foot among army officers, looking to the framing of a petition to Congress asking the appointment of dental surgeons in the army of the United States, it being probable that the naval service would also be included.

Dr. Davenport's paper on "Etiology–Spontaneous Abrasion" drew the fire of Dr. Chandler. Drs. Atkinson, Freidenburg and others took part. Dr. Barrett made some remarks on "Anæsthesia," Drs. Litch, Atkinson and others discussing it. Dr. Stockton of New Jersey presented a paper on the construction of artificial teeth. Dr. John Allen, of New York, read a paper on the "Relations of Dentistry to the Fine Arts." Dr. Grant, of Boston, read a paper on "Dental Prosthesis. This last speaker was a negro. His paper was well prepared; beside he would at times during its delivery set it aside to use the black-board or orally impress his points beyond what he had written; the applause showed how he was received. Dr. Flary read a paper on "The Present Status and Prospects of Mechanical Dentistry.

The election of officers resulted in no changes save the retirement of President Pierce and the election of Dr. H. A. Smith, of Cincinnati, Ohio.

Professor I. K. Buchanan, of this city, read a paper on "The Central Relations of the Teeth and Adjacent Parts to the Entire Constitution, through the Medium of the Proximate Portions of the Brain," and followed it by black-board explanations of great interest. Drs. Atkinson, Fellibrown, Portland, Me., following Dr. Brophy, of Chicago, read a paper on "Dental Education." It was urged that a higher plane must be attained by the dental practitioner. The fact is this paper urged very much what this journal hopes to be largely instrumental in bringing about, namely, a closer alliance and a better understanding between dental and medical schools (and that time's going to come). The Doctor urged further that the student should study the whole human system. That a dental student should be required to take a medical course first. Dr. Litch, of Philadelphia, and Professor Shepard, of Boston, followed in the pros and cons bearing on this paper. Dr. F. M. Odell, of this city, gave his own personal experience, both as medical and dental student.

The body adjourned to meet August, 1882, in the Queen City of the West, Cincinnati, Ohio. In conclusion, we are more and more convinced of the practical combination aimed at by our journal, namely, that of dental and medical reciprocity.

DR. WILLIAM FARMER, of Wyethville, Virginia, was our first caller in our new home, 20 Courtlandt St. The Doctor was in attendance at the Dental Convention, and from his constant and close attention we are satisfied will go back to the Dominion a wiser man. We hope any of our subscribers and friends who visit New York, will make the PRACTITIONER'S sanctum head-quarters, and feel perfectly at home. Our business manager is at your service in any way possible during your stay in this greatest city of the *whole world.*

